# Novel LC–MS/MS method for analysis of metformin and canagliflozin in human plasma: application to a pharmacokinetic study

**DOI:** 10.1186/s13065-019-0597-4

**Published:** 2019-07-09

**Authors:** Dalia Mohamed, Mona S. Elshahed, Tamer Nasr, Nageh Aboutaleb, Ola Zakaria

**Affiliations:** 1 Analytical Chemistry Department, Faculty of Pharmacy, Helwan University, Ein Helwan, Cairo, 11795 Egypt; 2 Pharmaceutical Chemistry Department, Faculty of Pharmacy, Helwan University, Ein Helwan, Cairo, 11795 Egypt; 3 Pharmaceutical Analytical Chemistry Department, Faculty of Pharmacy, October University for Modern Sciences and Arts, 6 October City, 11787 Egypt

**Keywords:** Metformin, Canagliflozin, HPLC–MS/MS, Human plasma, Pharmacokinetic study

## Abstract

Highly sensitive and selective liquid chromatography/tandem mass spectrometry (LC–MS/MS) method was developed and validated for the simultaneous estimation of the recently approved oral hypoglycemic mixture; metformin (MET) and canagliflozin (CFZ) in human plasma using propranolol HCl (PPL) and tadalafil (TDF) as internal standards (IS), respectively. Analytes were extracted using protein precipitation induced by acetonitrile then liquid–liquid extraction was performed using ethyl acetate. Reversed phase HPLC was carried out using C18 analytical column (50 mm × 4.6 mm i.d., 5 µm) with a simple isocratic mobile phase composed of 0.1% formic acid and acetonitrile (60:40, *v/v*). Detection was performed on a triple quadrupole mass spectrometer employing electrospray ionization technique, operating in multiple reaction monitoring (MRM), with the transitions of m/z 130.2 → 60.1, m/z 462.3 → 191.0, m/z 260.2 → 183.0 and m/z 390.2 → 268.2 for MET, CFZ, PPL and TDF, respectively, in the positive ion mode. The analysis was carried out within 5 min over a linear concentration range of 50–5000 ng/mL for MET and 10–1000 ng/mL for CFZ. The method was validated in accordance with the FDA guidelines for bioanalytical method. All obtained recoveries were higher than 90.0% while the accuracy was in the range of 88.14–113.05% and the relative standard deviation was below 10.0% for all investigated drugs by the proposed method. The achieved promising results has allowed for the successful application of the developed LC–MS/MS method to a pharmacokinetic study of the target drugs after their oral administration to Egyptian healthy volunteers. The pharmacokinetic study was accomplished after the agreement of the ethics committee.

**Electronic supplementary material:**

The online version of this article (10.1186/s13065-019-0597-4) contains supplementary material, which is available to authorized users.

## Introduction

Type 2 diabetes is a long-term metabolic disorder in which the body becomes resistant to the effects of insulin, a hormone that regulates sugar absorption [1]. At the present time it was evident that the oral hypoglycemic drugs that are usually recommended as mono-therapy are insufficient for hypoglycemic control of type-2 diabetic patients [2]. Thus, combination regimen including drugs with different and complementary mechanisms of action are suggested for accomplishing suitable blood glucose levels [3, 4].

**Fig. 1:**
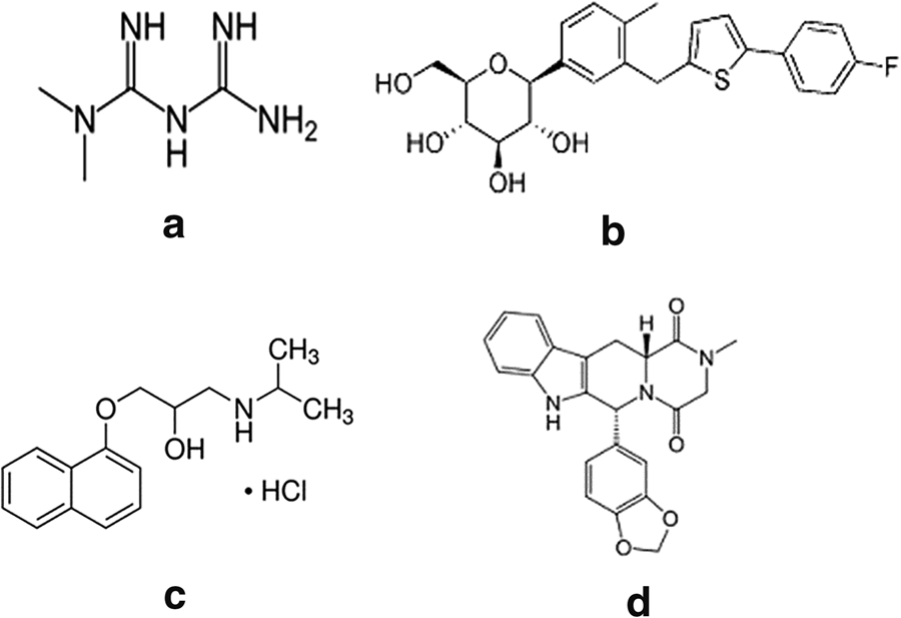
Chemical structures of: **a** metformin, **b** canagliflozin, **c** propranolol HCl and **d** tadalafil

Canagliflozin (CFZ); chemically named as (2S,3R,4R,5S,6R)-2-(3-{[5-(4-fluorophenyl)thiophen-2-yl]methyl}-4-methylphenyl)-6-(hydroxymethyl)oxane-3,4,5-triol [5] (Fig. 1) belongs to the sodium-glucose co-transporter (SGLT2) inhibitors group which is a new class of glucose lowering agents and is approved recently by food and drug administration [6]. SGLT2 inhibition blocks re-absorption of glucose, reduces the renal threshold for glucose, increases urinary glucose excretion thus lowering blood glucose through an insulin-independent mechanism [7, 8]. SGLT2 inhibitors can be used as mono-therapy or combined to any of the current classes of the glucose-lowering agents [9].

Metformin hydrochloride (MET) is an orally administered biguanide utilized for treating type 2 diabetes mellitus. It is chemically known as 1,1-Dimethylbiguanide monohydrochloride [10] (Fig. 1). MET lowers glucose by reducing hepatic glucose production and gluconeogenesis and by enhancing insulin sensitivity and peripheral glucose uptake into skeletal muscle [11, 12]. The mechanism of action involved is that it increases 5′adenosine monophosphate activated protein kinase enzyme activity which results in the stimulation of glucose disposal into skeletal muscle, fatty acid oxidation in muscle and liver, and the inhibition of liver gluconeogenesis, cholesterol and triglyceride synthesis and lipogenesis [13].

Analytical methods, including HPLC-UV [14], HPLC with fluorescence detection (HPLC-FLD) [15] and HPLC–MS/MS [16–19] were developed for the quantitative estimation of CFZ in different biological matrices. However, for the first two methods HPLC–UV [14], HPLC-FLD [15], the authors have performed their studies on spiked human plasma samples, the two methods were not extended to analyze CFZ from human volunteers or apply them to pharmacokinetics studies. Regarding the HPLC–MS/MS methods [16–19], they provide faster separation, more sensitive analysis and less mobile phase consumption. Actually, the concentration of CFZ in the plasma of patients from clinical trials was estimated by the LC–MS/MS methods [16–19] nevertheless, the different parameters of the assay were not fully disclosed. A pharmacokinetic study [20] was performed and fully validated according to the FDA [21] and EMEA [22] guidelines, however, the study was performed on rat plasma samples. Lately, a fully validated LC–MS/MS pharmacokinetic study was performed on healthy Indian male subjects [23].

As MET is released in the market long time ago, several methods could be found in the literature for its determination in different biological fluids either alone or combined with other drugs [24–34]. Regarding the HPLC–UV methods [24–28], they have suffered from certain disadvantages as lack of sensitivity, use of complicated sample preparation procedures as chemical derivatization, use of two step extraction, use of column-switching system or use of large sample volumes. As for the HPLC–MS/MS methods [29–34], they have provided higher sensitivity, selectivity and throughput. Some of the reported HPLC–MS/MS methods were extended to study the pharmacokinetics of MET in human volunteers.

Being a recently approved and released combination in the market, a sensitive and robust bioanalytical method is required for the estimation of CFZ and MET in samples of human plasma to provide pharmacokinetic information which would be beneficial for designing consequent clinical trials and suitable investigation in post-marketing clinical trials. The obtained results from the pharmacokinetic study could support the drugs interaction studies as well as therapeutic drug monitoring. Thus, in the present work, a novel, highly selective and sensitive LC–MS/MS method was developed and validated as per FDA guidelines [21] for the simultaneous determination of CFZ and MET in human plasma and applied to a pharmacokinetics study in Egyptian healthy volunteers after oral administration of the studied drugs in dosage form.

## Experimental

### Materials

Metformin hydrochloride (certified purity 100.4%) and propranolol (certified purity 99%) (Fig. 1) were obtained from Sigma Aldrich (Darmstadt, Germany). Canagliflozin (certified purity 99.1%) was purchased from Beijing Huikang Boyuan Chemical Technology Co. Ltd. (Beijing, China). Tadalafil (certified purity 99.9%) (Fig. 1) was supplied by SMS Pharmaceuticals ltd., India.

Human blank plasma was purchased from the “Holding Company for Biological Products and Vaccines (VACSERA)”, Egypt. Both methanol and acetonitrile (≥ 99.9%) of HPLC grade were purchased from Sigma Aldrich (Darmstadt, Germany) while formic acid (98–100% extra-pure) and ethyl acetate (HPLC grade 99.8%) were obtained from Scharlau (Barcelona, Spain). Vokanamet^®^ tablets (B.N EGZT300) comprising 850 mg metformin hydrochloride and 50 mg canagliflozin were obtained through an online Canadian pharmacy.

### Equipment

Chromatographic analysis was carried out using an Agilent1260 HPLC system (Agilent Technologies, USA). Detection by mass spectrometry was carried out using a triple quadrupole API 4000 (ABSciex, Canada), using positive electrospray ionization (+ESI) and multiple reaction monitoring (MRM) mode. Control of hardware as well as acquisition of the data were done utilizing an Analyst 1.6.3 Software (ABSciex, Canada).

### Conditions of liquid chromatography and mass spectrometry

Separation was achieved on Zorbax C18 (50 mm × 4.6 mm, 5 μm) analytical column (Agilent, USA) maintained at ambient temperature. The mobile phase used was acetonitrile: 0.1% formic acid (40:60, *v/v*) delivered at a flow rate of 0.5 mL/min with an injection volume of 2 μL. The total LC run time was 5 min.

The mass spectrometer was operated +ESI mode. Quantification was accomplished through using multiple reaction monitoring (MRM) of the transitions of mass-to-charge ratio m/z 130.2 → 60.1, m/z 462.3 → 191.0, m/z 260.2 → 183.0 and m/z 390.2 → 268.2 for MET, CFZ, PPL and TDF, respectively. The following parameters were set: Air (zero grade) as nebulizer gas, nitrogen as auxiliary, curtain and collision gas, curtain gas = 10 psi, collision gas = 10 psi, ion spray temperature = 400 °C, ion spray voltage = 2000 V, ion source gas one and ion source gas two = 25 and 45 psi, respectively.

### Stock and working solutions

Standard stock solutions were performed in 100-mL volumetric flask through the dissolving of 50 mg MET and 10 mg CFZ separately in 30 mL methanol then diluting to volume with methanol too, thus, obtaining a final concentration of 500 μg/mL and 100 μg/ml for MET and CFZ, respectively.

Two standard working solutions were prepared. For the first working solution (WS 1), an aliquot of 10 mL of the stock solution was transferred to 100-mL volumetric flask and the volume was completed using methanol, thus, obtaining a concentration of 50 μg/mL MET and 10 μg/mL CFZ. The second working solution (WS 2) was prepared by diluting 10 mL of (WS 1) with methanol in 50-mL volumetric flask, thus, obtaining a concentration of 10 μg/mL MET and 2 μg/mL CFZ. The solutions were then successively diluted with methanol to prepare working solutions in the concentration range 500–50,000 ng/mL for MET and 100–10,000 ng/mL for CFZ. All stock solutions and working solutions were stored at 2–8 °C.

### Internal standard solutions

Two internal standards were used, PPL and TDF for MET and CFZ, respectively. Solutions of 100 μg/mL PPL and 700 μg/mL TDF were prepared in 100-mL volumetric flask using methanol as the solvent.

### Calibration standards and quality control (QC) samples

Calibration curves were prepared by spiking 50 μL of increasing concentrations of either MET or CFZ from their working standard solutions and 50 μL of IS into 400 μL blank human plasma. Calibration standards were made at concentration in the range of 50–5000 ng/mL for MET and 10–1000 ng/mL for CFZ. The plasma calibration curve comprised a blank sample (matrix sample excluding the internal standards), a zero sample (matrix sample including the internal standards), and non-zero samples within the probable range, comprising the lower limit of quantification (LLOQ).

Quality control samples were prepared at 50 and 10 ng/mL (lower limit of quantification quality control, LLOQ), 150 and 30 ng/mL (low quality control, LQC), 1000 and 200 ng/mL (middle quality control, MQC) and 4000 and 800 ng/mL (high quality control, HQC) for MET and CFZ, respectively. The spiked plasma samples (calibration standards and QCs) were treated according to the sample preparation described below.

### Extraction protocol

The plasma samples were spiked with the drugs and internal standards, then, protein precipitation was achieved through adding 1 mL acetonitrile, followed by liquid–liquid extraction using 3 mL ethyl acetate. Vortex mixing was performed for 4 min then centrifugation was done at 4000 rpm at 10 °C for another 5 min. The clear supernatant was relocated carefully to a Wassermann tube and concentrated at 60 °C then reconstituted with methanol. Finally 2 µL aliquot was injected into the LC–MS/MS system.

## Bio-analytical method validation

The FDA guidelines for the bioanalytical method [21] were followed for complete validation of the developed method through calculating all the validation parameters as follows:

### Selectivity

The selectivity of the method was tested by screening six different batches of blank human plasma. The peak area of blank plasma samples were measured and compared to a set of samples of blank plasma spiked with the investigated analytes at their LLOQ levels. The LLOQ was considered as the lowest concentration of each investigated analyte which can be quantitatively estimated with satisfactory precision and accuracy.

### Linearity and range

The linearity of the method was judged by means of a blank sample (matrix sample excluding the internal standards), a zero sample (matrix sample including the internal standards) and eight non-zero samples. Peak area ratios of each drug to the IS were plotted versus the standard concentrations, the linearity was assessed by linear regression analysis and the concentration of the analytes were estimated from calibration curves. For each calculated standard concentration; deviation from the nominal value was permitted up to ± 15% except for LLOQ where deviation was acceptable up to ± 20%.

### Accuracy and precision

Inter-day and intraday accuracy and precision were evaluated at four dissimilar concentrations levels (LLOQ, LQC, MQC and HQC) using six replicates for both analytes. The accuracy and precision were determined and expressed in terms of percentage accuracy and coefficient of variation (RSD%), respectively. For satisfactory accuracy and precision, variation was acceptable up to ± 15%, except for LLOQ where the value of the variation was considered suitable up to ± 20%.

### Recovery

The recovery of the studied drug by the proposed method was calculated through the comparison of the peak areas of the analytes that were spiked into blank plasma which was exposed to the whole extraction procedure at the three QC levels (LQC, MQC and HQC) with the peak areas of the analytes in post-extracted plasma samples. It is not a requirement that the recovery of the analyte is 100%, however, it is necessary that the variability is consistent, precise, and reproducible among different samples.

### Matrix effects

The matrix effect was investigated utilizing blank plasma of six different batches. The ratio of the peak area of blank samples in which the matrix was spiked with the investigated analytes after the extraction procedure to the peak area of pure analytes solution was calculated for each batch. The calculated RSD% should not be greater than 15%. This determination was performed at two different concentration levels (LQC and HQC).

### Dilution integrity

Dilution integrity was demonstrated through spiking the matrix with a concentration of the analytes above their HQC, then diluting this sample with the blank matrix. Dilution of samples should not affect the accuracy and precision. Accuracy and precision should be within ± 15%.

### Stability experiments

Stability experiments were accomplished in order to assess the stability of the analytes in plasma samples under several conditions which simulate the conditions that could occur during sample analysis. Short term stability, freeze and thaw stability, long term stability, auto sampler (processed sample) stability and dry extract stability were done at LQC and HQC levels utilizing six replicates from each level.

*Short term stability* was studied by examining samples that were defrosted at room temperature then left for 6 h before carrying out the analysis.

*Freeze*–*thaw stability* was examined by investigating the QC samples’ stability through four freeze–thaw cycles after being kept to freeze for 24 h. Samples were then thawed unassisted at room temperature for 2 h or even more then kept to freeze again at − 86 °C overnight for every freeze–thaw cycle.

*Long term stability* was studied, where samples were left at − 86 °C and examined by the end of this study (30 days). This period exceeds the time from the collection of the sample to the time of processing and analysis.

*Processed sample stability* was evaluated by leaving processed QC samples in the auto-sampler at 25 °C for 1 day (24 h) followed by analysis. This study is done to find out the consequences of an infrequent delay in the injection of extracted sample on the analyte stability.

*Dry extract stability* was assessed by processing QC samples that are kept as dry residues at room temperature without reconstitution. The samples were then reconstituted after 24 h and analyzed concurrently with a newly prepared calibration curve.

The stability of the QC samples were investigated by comparing their recoveries under the different stability conditions with those of freshly prepared samples. Samples were considered to be stable if the mean concentration at each QC level was within acceptable limits (± 15%) with RSD% not exceeding 15%.

## Application to pharmacokinetic study

After applying the full validation scheme, the developed method was utilized for the quantitation of the target analytes in plasma samples of 2 healthy Egyptian volunteers under fasting conditions. The volunteers were informed about the purposes and the probable hazards of this study and they were asked to sign a written consent where the study protocol was accepted by the institutional ethics committee. A pharmacokinetic study was performed where blood samples were collected after the oral administration of a dose equivalent to 850 mg MET and 50 mg CFZ at zero time and 0.5, 1, 1.5, 2, 3, 4, 6, 7, 8, 10, 12, 16, 24, 48 and 72 h. Then centrifugation of the samples was performed immediately at 3500 rpm for 10 min using ethylenediaminetetraacetic acid as an anticoagulant. The plasma samples were separated and prepared as described and kept frozen at − 86 °C till the analysis day. The plasma concentration–time curves were constructed and various pharmacokinetic parameters were calculated.

## Results and discussion

### Method development

Regarding the extraction procedure; both liquid–liquid extraction (LLE) and solid-phase extraction (SPE) are the techniques that are frequently utilized in the preparation of biological samples due to their influence on improving the sensitivity and robustness of the analysis method. However, MET is a highly polar compound, thus, it was extremely difficult to be extracted from the biological fluids utilizing the LLE technique. On the other hand, it was not wise to use SPE as it is considered an expensive technique, specifically in a high throughput analysis comprising a lot of samples. Accordingly, in the current study, a simple protein precipitation technique was established for the extraction of MET using acetonitrile which showed a higher efficiency of precipitation of protein with minimum loss of the extracted drug when compared to other solvents as methanol and acetone. However, protein precipitation only was inadequate for CFZ extraction which is more non polar if compared to MET. Furthermore, CFZ has high protein binding nature [8], therefore, LLE is recommended for the extraction of CFZ where best extraction was demonstrated using ethyl acetate. Consequently, we have combined both extraction techniques; protein precipitation using acetonitrile followed by LLE using ethyl acetate, then evaporation of the supernatant and reconstitution with methanol was performed which has resulted in clear extracts with the least matrix effect and the best quantitative extraction of the investigated analytes and IS.

**Fig. 2:**
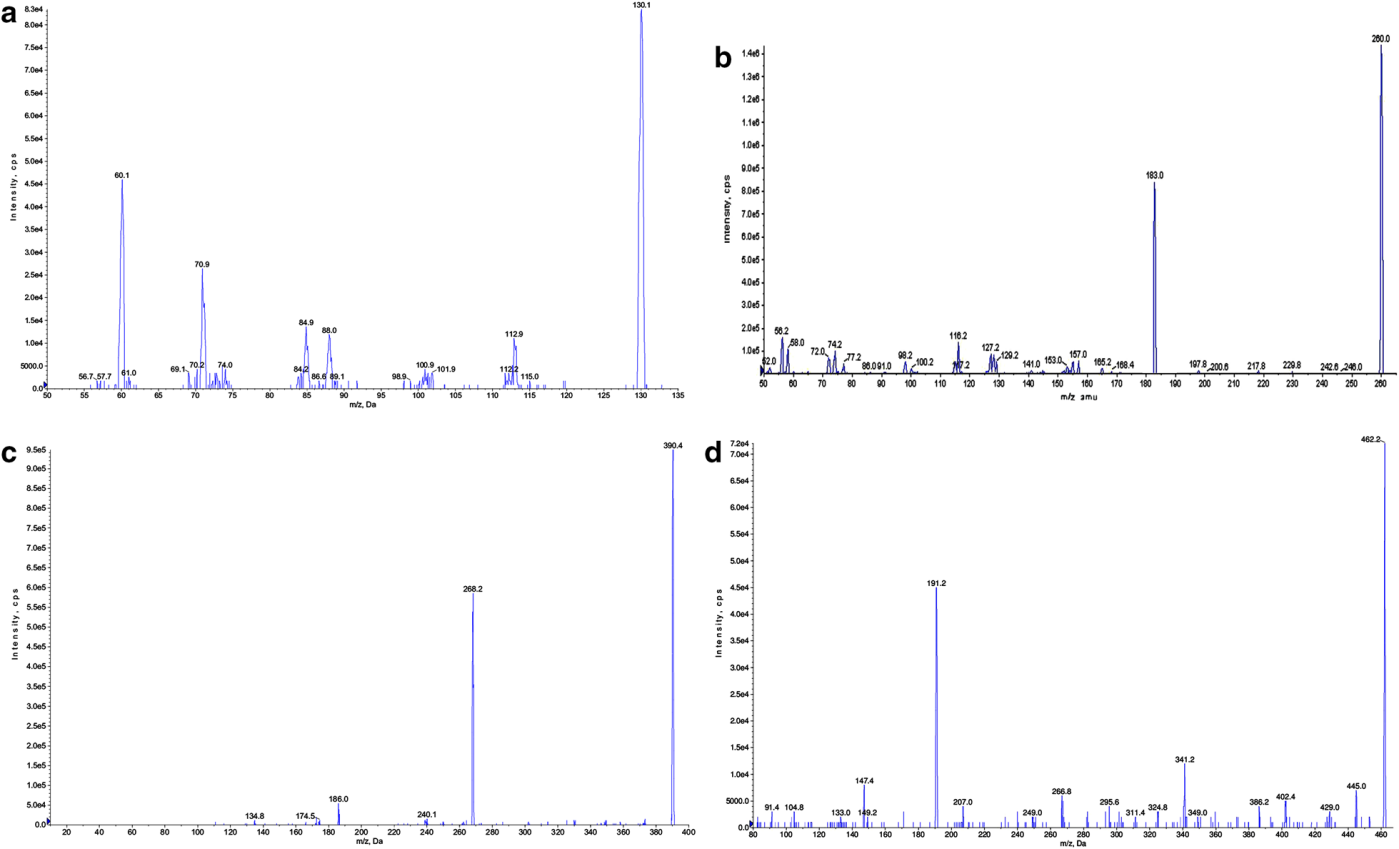
Product ion spectra of [M + H]^+^ of: **a** metformin, **b** propranolol HCl, **c** tadalafil and **d** [M + NH_4_]^+^of canagliflozin

Coupling of liquid chromatography to MS/MS detection is an extremely selective technique which results in insignificant interference of endogenous impurities. LC-MRM is an influential method specifically for pharmacokinetic studies as it offers the required sensitivity and the selectivity. In the MRM mode; the ions that are derived from the target analytes are the only monitored ions, therefore this technique was chosen for the present method development. Both +ESI and −ESI were investigated and it was obvious that the signal intensities acquired from the +ESI were higher than those acquired from the −ESI which could be attributed to the capability of the target analytes as well as the ISs to gain protons. In the Q1 MS full scan spectra; MET, PPL and TDF showed predominant protonated [M +H]^**+**^ parent ions at m/z 130.2, 260.2 and 390.2 ions, respectively, while CFZ gave [M + NH_4_]^**+**^ at m/z 462.3 as strong signal instead of the protonated molecule (which is commonly observed with neutral compounds [15]). The ammonium adduct ions [M + NH_4_]^+^ in positive ionization were previously detected for the estimation of CFZ in plasma [20]. As the details of the fragmentation patterns of MET [34] and CFZ [20] were previously discussed, thus, we are not demonstrating the data related to this. The most abundant ions found in the product ion mass spectrum were m/z 60.1, 191.0, 183.0 and 268.2 for MET, CFZ, PPL and TDL, respectively, as shown in Fig. 2. The optimized mass spectrometric parameters are abridged in Table 1.

**Table 1:** LC–MS/MS parameters selected for the quantification of metformin and canagliflozin using propranolol and tadalafil as internal standards

Analyte	Q1 (m/z)	Q3 (m/z)	DP (v)	EP (v)	CE (v)	CEP (v)
Metformin	130.2	60.1	26	10	19	12
Canagliflozin	462.3	191.2	61	10	15	18
Propranolol HCl	260.2	183.0	120	10	28	12
Tadalafil	390.2	268.2	73	10	50	17.4

Q1: precursor ion; Q3: product ion; DP: declustering potential; EP: entrance potential; CE: collision energy; CEP: cell exit potential

**Fig. 3:**
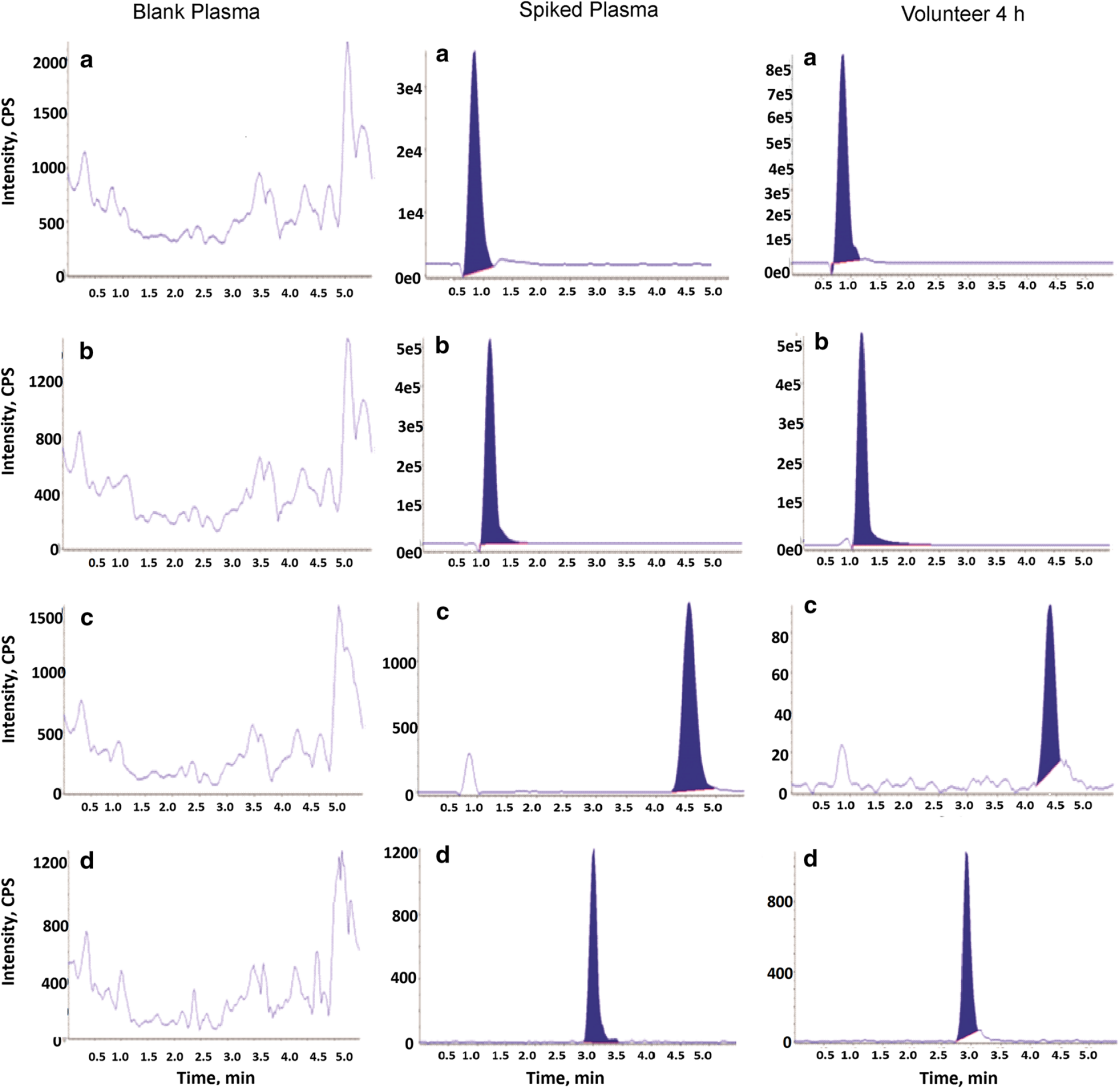
Mass chromatograms of blank plasma, plasma spiked with **a** metformin at LLOQ, **b** propranolol HCl, **c** canagliflozin at LLOQ and **d** tadalafil and plasma from volunteers 4 h after administration of one Vokanamet^®^tablet

MET and CFZ have dissimilar physicochemical properties, consequently, considerable effort was accomplished to adjust the chromatographic conditions in order to achieve sharp peaks shape and satisfactory response. The adjustment of the method included; mobile phase, flow rate, stationary phase and injection volume. Several trials with different columns were performed aiming at the optimization of the chromatographic conditions. Using Zorbax C18 column at room temperature with a flow rate of (0.5 mL/min) and very small injection volume (2 µL) has resulted in the highest chromatographic performance with the least solvent consumption. Methanol and acetonitrile as organic modifiers were examined in different ratios with formic acid. The use of acetonitrile rather than methanol has allowed for better response and elution of the two analytes in a short time. It was observed that the ratio of acetonitrile and 0.1% formic acid (40:60) as the mobile phase was the most suitable for obtaining the best sensitivity, efficiency and peak shape (Fig. 3).

### Method validation

#### Selectivity

Typical chromatograms of blank plasma, plasma spiked with MET, CFZ and ISs and plasma from a volunteer 4 h after the administration of one tablet containing MET (850 mg) and CFZ (50 mg) are demonstrated in Fig. 3. The retention time for MET and its IS is at 0.9 and 1.1 min while the retention time for CFZ and its IS is at 4.5 and 3 min. It was obvious that there was no significant interference from any endogenous plasma components at the retention time of the investigated analytes or IS.

#### Linearity and range

The calibration curves were found to be linear in the range of 50–5000 ng/mL for MET and 10–1000 ng/mL for CFZ. The blank and zero samples were used in order to verify the absence of interference but was excluded from the regression analysis. The regression equations were as follows:

For MET, Y = 0.0016X − 0.0088, r = 0.9971 and SD = 6.372

For CFZ, Y = 0.0056X − 0.0036, r = 0.9971 and SD = 6.337

Y = Peak area ratio of the analyte to the internal standard, X = Concentration of the analyte in ng/mL.

The r values, slopes and intercepts were calculated utilizing the linear regression analysis. All back-calculated concentrations were within ± 15% from the nominal concentrations. The LLOQ was determined as the lowest concentration of analyte (at S/N ratios of 10) which can be estimated quantitatively with satisfactory precision and accuracy (RSD < 20%).

#### Accuracy and precision

Intraday accuracy and precision were estimated through analyzing the samples (six replicates) in the same day, whereas inter-day was assessed through repeated analysis over 3 days. The examination of the spiked plasma samples has revealed that intraday accuracy of the assay has varied between 91.7 and 101.44% with a precision (RSD%) in the range of 2.98–8.465% for MET, while for CFZ, the inter-day accuracy has varied between 90.65 and 113.05% with a precision (RSD%) of 1.92–7.085% (Table 2). The between-run accuracy was within the range of 93.36–107.44% with RSD% of 4.98–8.275% for MET and 88.14–111.22% with RSD% of 4.53–10.24% for CFZ (Table 2).

**Table 2:** Intra-day and inter-day precision and accuracy for the determination of metformin and canagliflozin in human plasma

Studied drug	QC level	Intra-day, n = 6			Inter-day, n = 6 × 3		
		Mean conc. found (ng/ml)	Accuracy%	RSD%	Mean conc. found (ng/ml)	Accuracy%	RSD%
Metformin	LLOQ (50 ng/mL)	47.24	94.48	7.301	48.55	97.10	8.275
	LQC (150 ng/mL)	152.17	101.45	2.980	161.15	107.43	4.980
	MQC (1000 ng/mL)	943.81	94.38	8.465	935.77	93.58	6.347
	HQC (4000 ng/mL)	3667.95	91.70	4.114	3734.26	93.36	6.626
Canagliflozin	LLOQ (10 ng/mL)	11.31	113.10	1.921	11.12	111.20	4.528
	LQC (30 ng/mL)	32.73	109.10	2.173	29.95	99.83	10.244
	MQC (200 ng/mL)	181.30	90.65	7.085	176.28	88.14	4.664
	HQC (800 ng/mL)	768.06	96.01	6.023	738.32	92.29	6.644

n = 3 days, 6 replicates per day

#### Recovery

The recoveries of MET, CFZ and IS were calculated at the three QC levels (six replicates). As the recovery describes the efficiency of the separation of analytes from the samples, thus, the results shown in Table 3 prove the efficiency of the extraction protocol introduced by the proposed method where the recoveries of the analytes were satisfactory and consistent. The mean of the recovery values were 98.21% and 94.32% with precision (RSD%) 4.55 and 5.67 for MET and CFZ, respectively.

**Table 3:** Recovery data and matrix effect for the determination of metformin and canagliflozin by the proposed method in human plasma

	QC level	Metformin		Canagliflozin	
		Recovery%	RSD%	Recovery%	RSD%
Recovery data	LQC	98.55	3.379	90.12	3.776
	MQC	97.69	6.766	94.04	7.149
	HQC	98.40	3.493	98.79	6.077
Matrix effect	LQC	98.43	6.075	95.95	13.136
	HQC	96.26	4.083	104.98	5.887

Mean percentage recovery and RSD were calculated using six lots of plasma samples

#### Matrix effect

The matrix effect defines the efficiency of the ionization of the analytes in the ion source and whether it is affected by the co-eluting matrix constituents. The matrix effect was examined for MET, CFZ and IS in two QC levels (LQC and HQC). The data abridged in Table 3 has indicated that there was no significant matrix effect on the ionization (suppression or enhancement) of the analytes which proves that the utilized conditions for sample processing has efficaciously removed any probable interference from the matrix.

#### Dilution integrity

Dilution integrity is done to check if samples’ dilution would interfere with the accuracy and precision of results [22]. To study dilution integrity, quality control samples (two and four times the HQC concentration) were prepared and diluted by factor of 2 and 4 with blank matrix. Accuracy values for dilution integrity were found to be 92.40 and 92.68% for MET and 101.68 and 106.90% for CFZ. While RSD% were 4.74 and 6.44% for MET and 5.56 and 4.09% for CFZ.

#### Stability

Different stability experiments, short term stability, processed sample stability, repeated freeze–thaw cycles, dry extract stability and long term stability showed that the mean% nominal values of the analytes were within ± 15% of the predicted concentrations for the analytes at their LQC and HQC levels. The results demonstrated in Table 4, were all within the acceptable limits and proved the good stability of MET and CFZ.

**Table 4:** Results of stability tests under different conditions for the determination of metformin and canagliflozin QC samples by the proposed LC–MS/MS method

	Canagliflozin				Metformin			
	Spiked conc. (ng/mL)	Mean found (ng/mL)	Accuracy%	RSD%	spiked conc. (ng/mL)	Mean found (ng/mL)	Accuracy%	RSD%
Short term stability	30	26.30	87.67	1.90	150	132.38	88.25	1.69
	800	704.72	88.09	2.66	4000	3565.06	89.13	2.57
Freeze and thaw stability	30	28.61	95.37	6.36	150	141.10	94.07	3.76
	800	713.42	89.18	3.52	4000	3552.69	88.82	2.29
Dry extract stability	30	28.54	95.13	4.54	150	139.01	92.67	7.34
	800	696.46	87.06	1.20	4000	3699.81	92.50	3.26
Processed sample stability	30	28.12	93.73	3.11	150	134.03	89.35	1.70
	800	698.63	87.33	0.61	4000	3836.46	95.91	6.01
Long term stability	30	29.77	99.23	7.73	150	130.4	86.93	1.87
	800	687.82	85.98	0.83	4000	3619.81	90.50	4.63

Mean, accuracy% and RSD% were calculated using three determinations

## Pharmacokinetic application

**Fig. 4:**
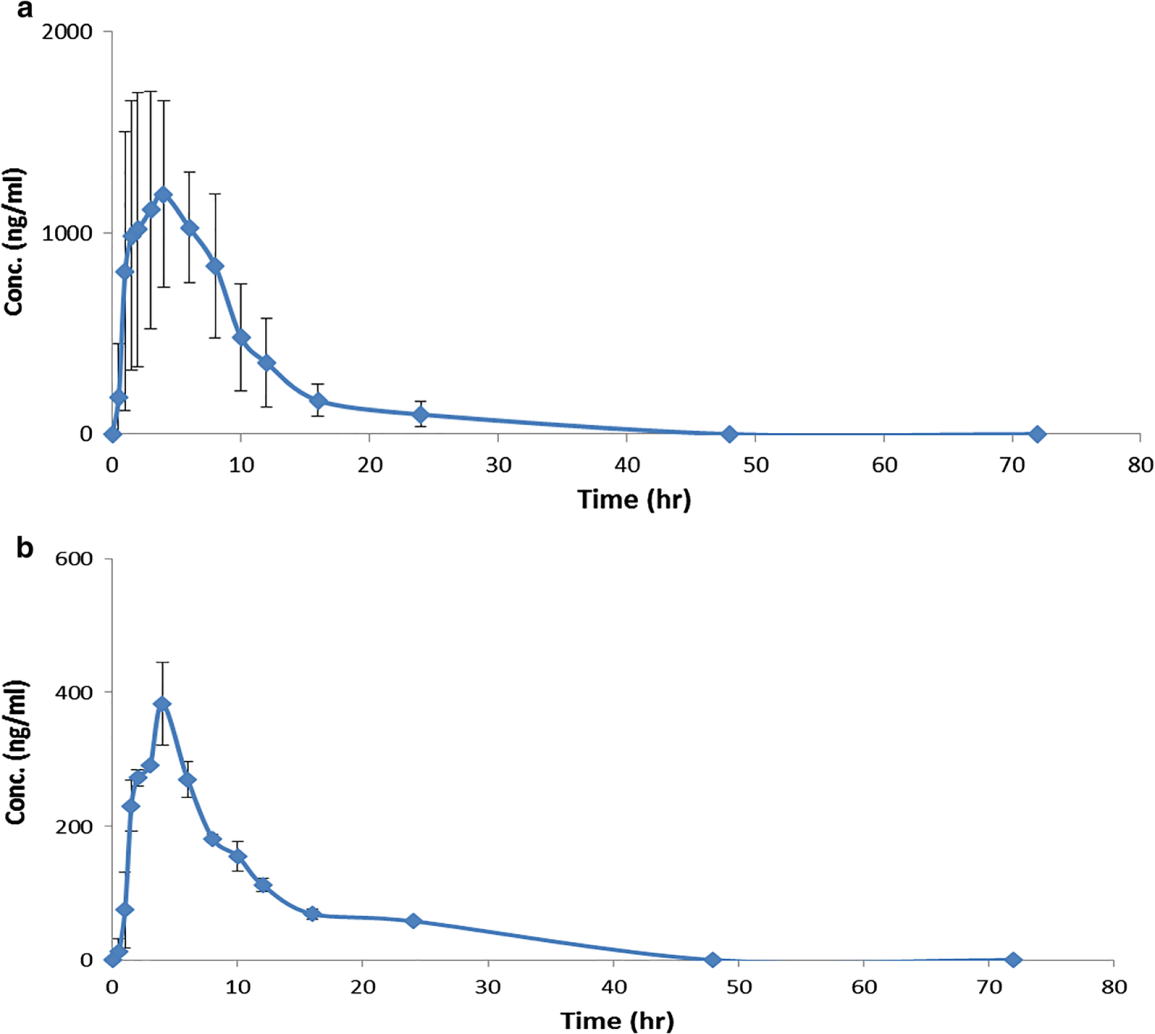
Mean plasma concentration–time profile of: **a** metformin and **b** canagliflozin in human plasma following oral dosing of one Vokanamet^®^ tablet to healthy volunteers

The proposed method was effectively utilized in a pharmacokinetic study of MET and CFZ after the oral administration of their combined dosage form; Vokanamet^®^ tablets in healthy Egyptian volunteers. Fasting of the volunteers has removed the possible interaction from the intake of food or caffeine. In accordance with the “Egyptian Ministry of Heath” and “October University for Modern Sciences and Arts University” research ethics rules, the approval of the current study by the ethics committee was obligatory (A1/E1/2017 PD). The study was designed complying with the FDA guidelines [21]. The investigated pharmacokinetic parameters encompassed C_max_, t_max_, AUC_0−t_, AUC_0−**∞**_, half-life (t_1/2_) and elimination rate constant (K_el_). Mean plasma concentration–time curves of MET and CFZ in a single dose study are illustrated in Fig. 4. The pharmacokinetic parameters results are presented in Table 5.

**Table 5:** Pharmacokinetic parameters for metformin and canagliflozin following oral administration of one Vokanamet^®^ nominally containing 850 mg of metformin and 50 mg of canagliflozin

PK parameter	Metformin	Canagliflozin
t_max_ (h)	3.5 ± 0.71	4 ± 0.0
C_max_ (ng/mL)	1197.50 ± 470.23	383.13 ± 62.41
AUC_0−t_ (ng h/mL)	11849.18 ± 5871.57	3409.06 ± 208.50
AUC_0−∞_ (ng h/mL)	12575.62 ± 6220.67	3962.65 ± 223.77
*t*_1/2_ (h)	6.03 ± 0.62	8.19 ± 0.06
K_el_ (h^−1^)	0.12 ± 0.01	0.08 ± 0.01

## Conclusion

A novel HPLC–MS/MS method was established and validated for the estimation of metformin and canagliflozin (recently approved oral hypoglycemic mixture) simultaneously in human plasma. The results of the developed method were satisfactory and they prove its selectivity, accuracy and precision. Reliable and reproducible recoveries were obtained for the analytes and IS from human plasma, with negligible interference from the matrix. Owing to the LLOQ, the proposed method was effectively utilized to estimate plasma concentrations of the studied drugs in a pharmacokinetic study involving Egyptian healthy volunteers. The studied pharmacokinetic parameters could be compared later on with the results obtained from other ethnic population.

## Additional files

**Additional file 1.** Chromatograms of increasing concentrations of calibration curve of metformin.

**Additional file 2.** Chromatograms of increasing concentrations of calibration curve of canagliflozin.

## Data Availability

The data supporting the findings are uploaded in the Additional files 1 and 2.

## Abbreviations

% RSD: percentage relative standard deviation
CFZ: canagliflozin
EMEA: European Medicines Agency
FDA: food and drug administration
h: hour
HPLC: high performance liquid chromatography
HPLC-FLD: high performance liquid chromatography with fluorescence detection
HPLC–MS/MS: high performance liquid chromatography tandem mass spectrometry
HPLC–UV: high performance liquid chromatography with ultraviolet detection
HQC: high quality control
i.d.: internal diameter
IS: internal standard
LLE: liquid liquid extraction
LLOQ: lower limit of quantification
LOD: limit of detection
LOQ: limit of quantitation
LQC: low quality control
m/z: mass to charge ratio
MET: metformin
mg: milligram
min: minute
mL: milliliter
mm: millimeter
MQC: middle quality control
MRM: multiple reaction monitoring
ng: nanogram
PPL: propranolol HCl
QC: quality control
r: correlation coefficient
rpm: rotation per minute
SD: standard deviation
TDF: tadalafil
μg: microgram
μL: microliter
μm: micrometer
