# Supplementary figures and images for: Novel LC–MS/MS method for analysis of metformin and canagliflozin in human plasma: application to a pharmacokinetic study
Source: BMC Chem. 2019 Jul 9;13(1):82. doi: 10.1186/s13065-019-0597-4 (PMC6661773; doi:10.1186/s13065-019-0597-4)

## Propranolol

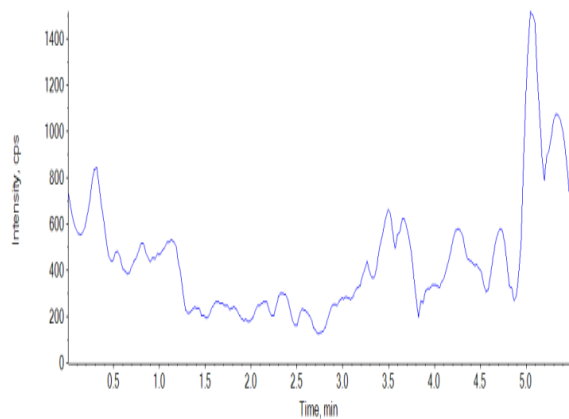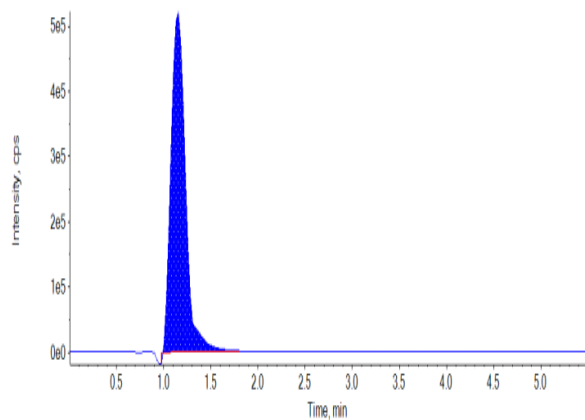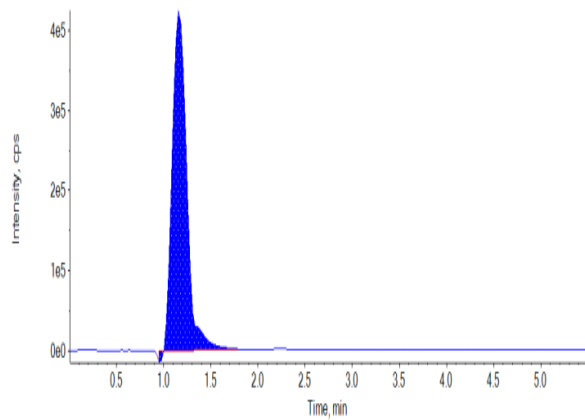

## Metformin

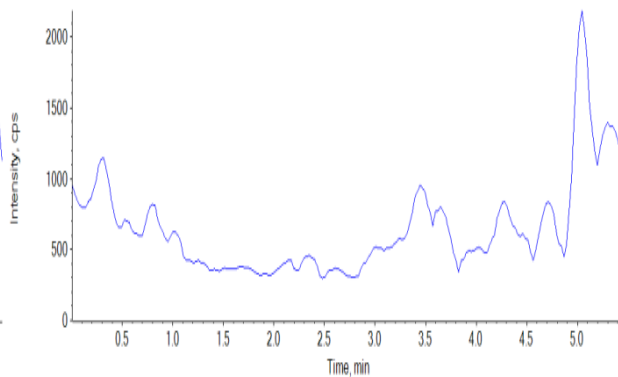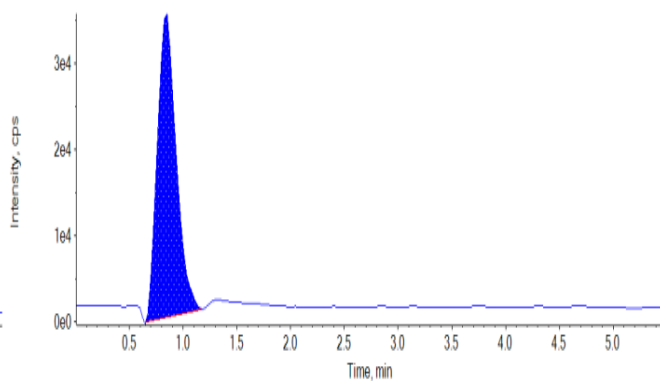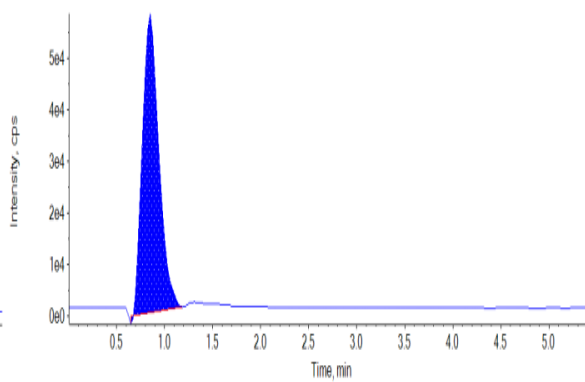

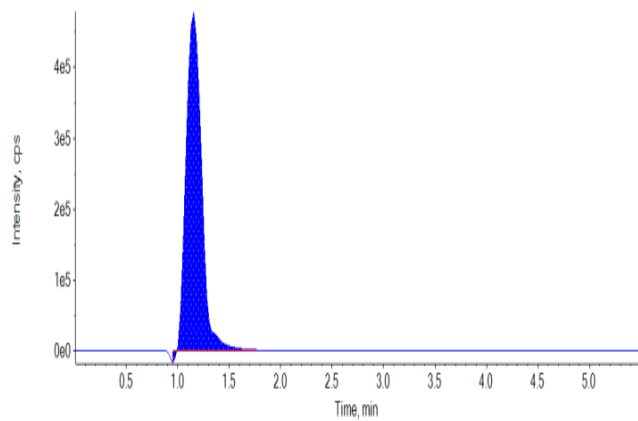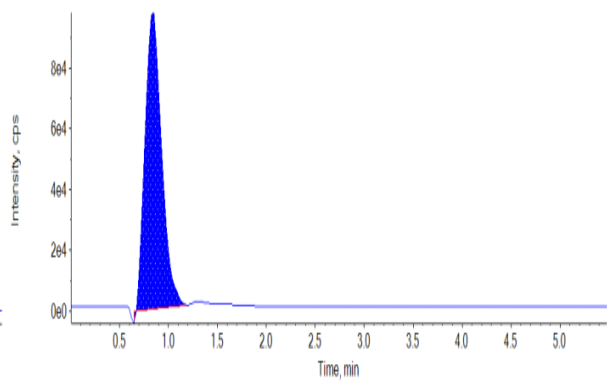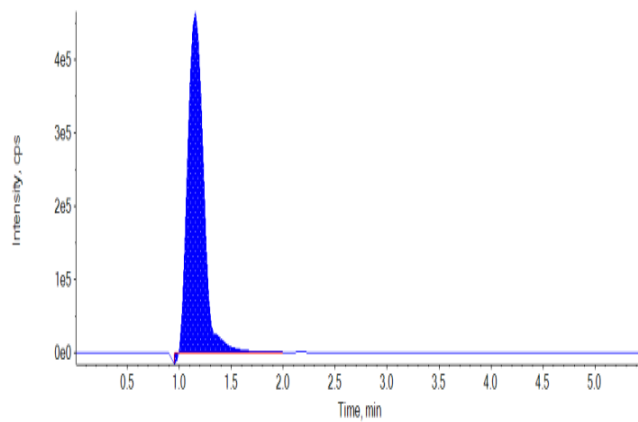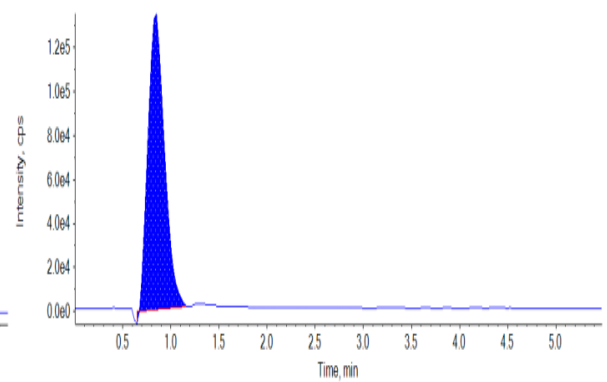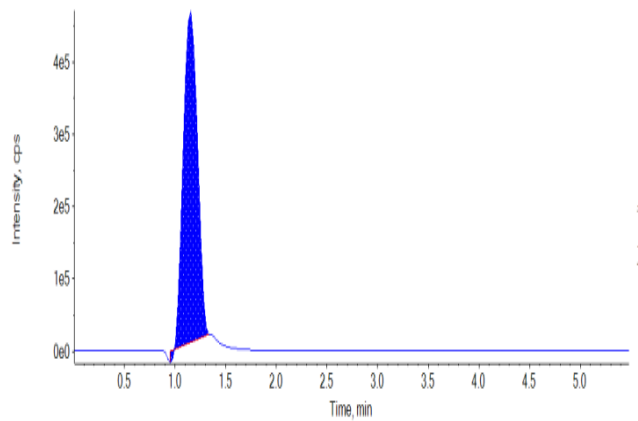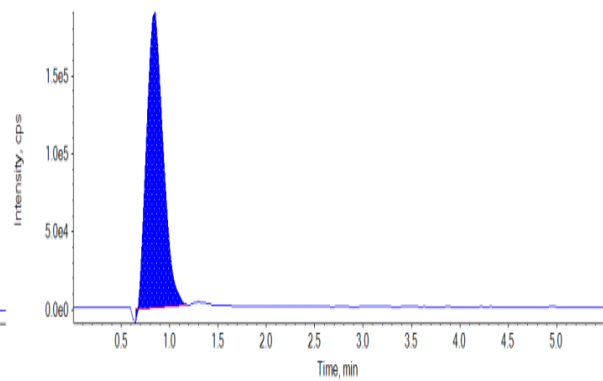

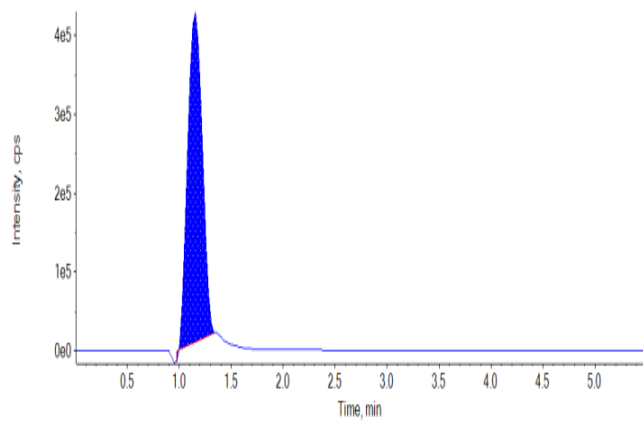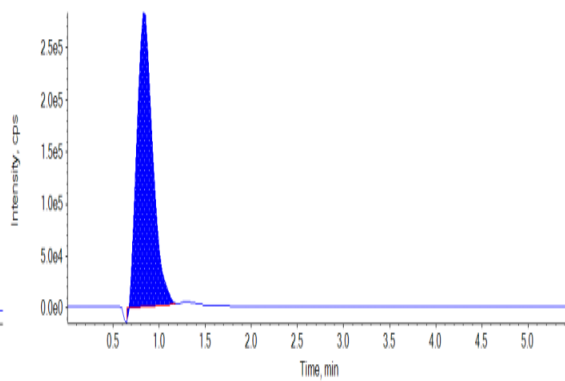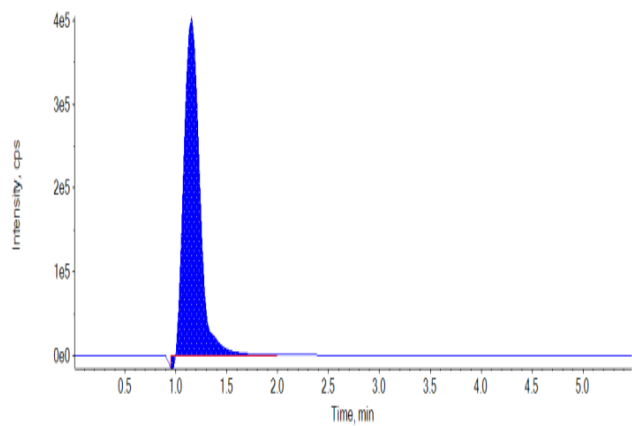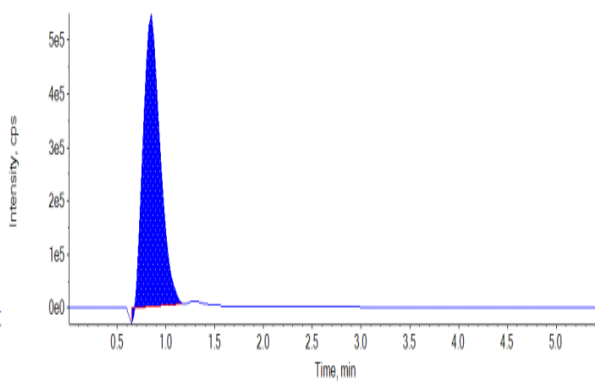

Supplement: Supplementary file 1 — Additional file 1. Chromatograms of increasing concentrations of calibration curve of metformin. [file 13065_2019_597_MOESM1_ESM.pdf]

## Tadalafil

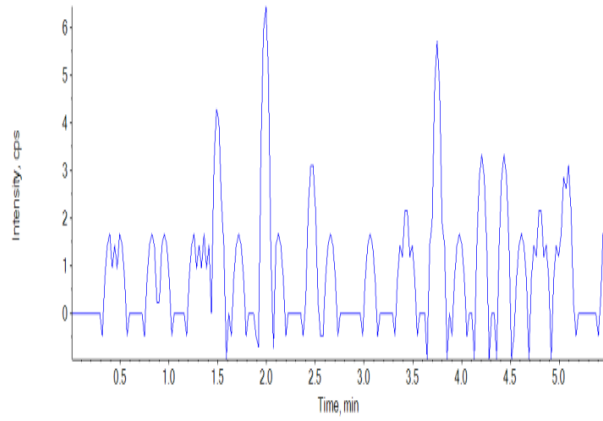

## Canagliflozin

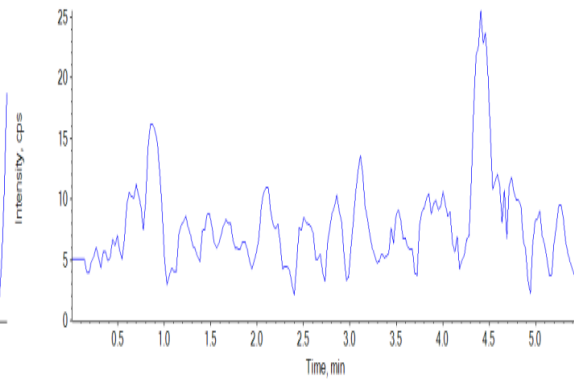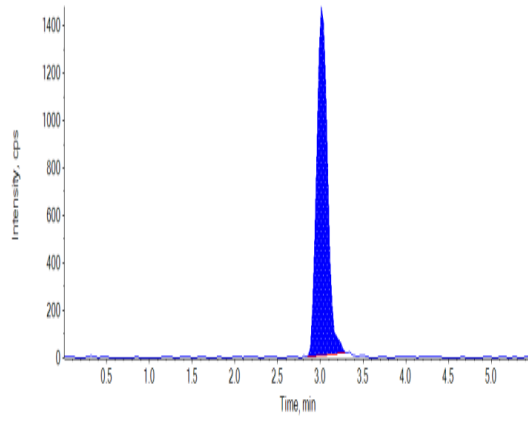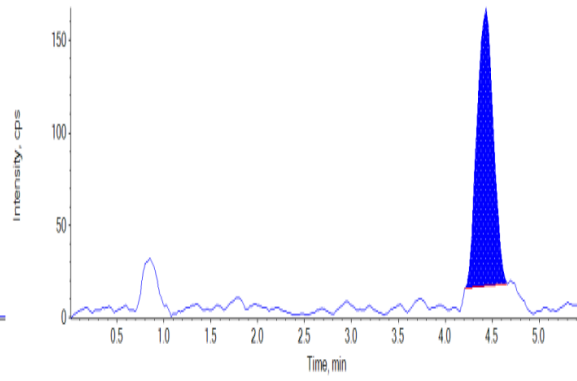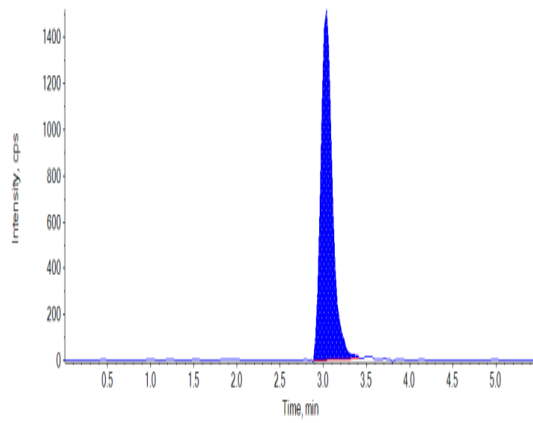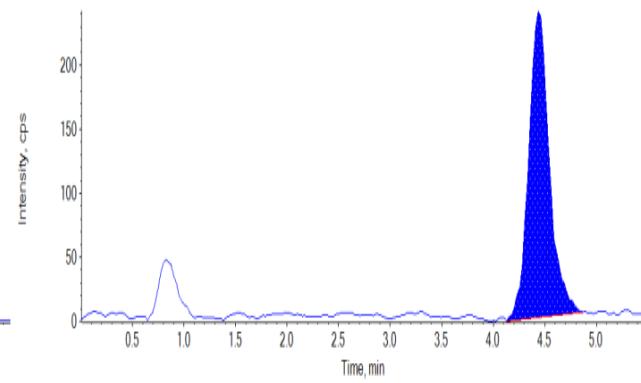

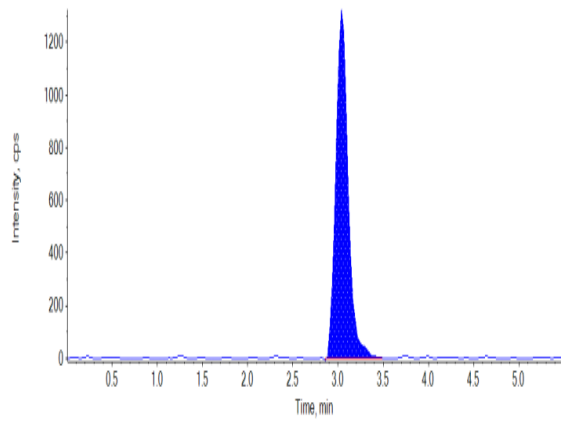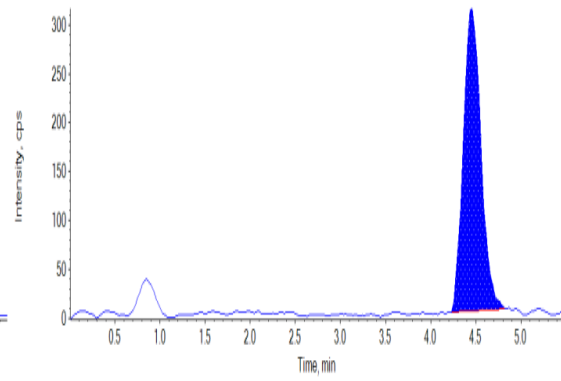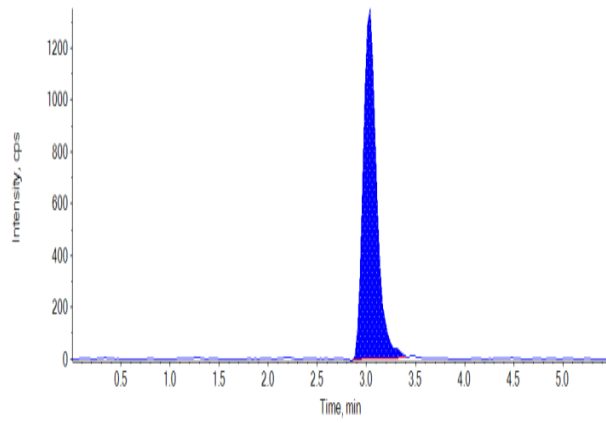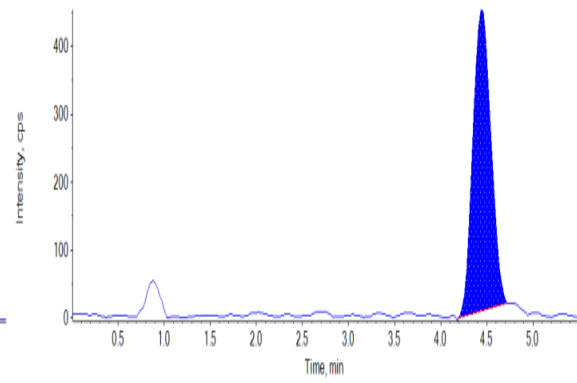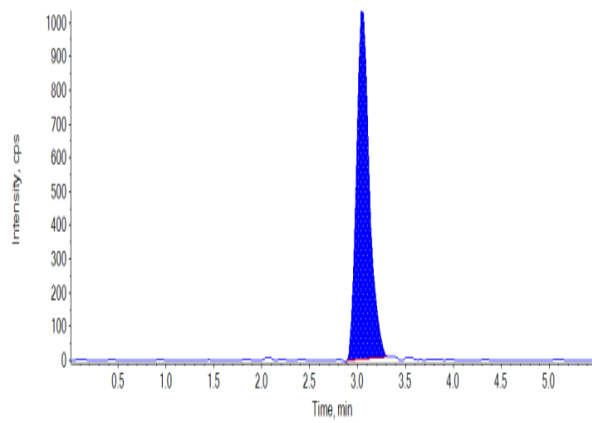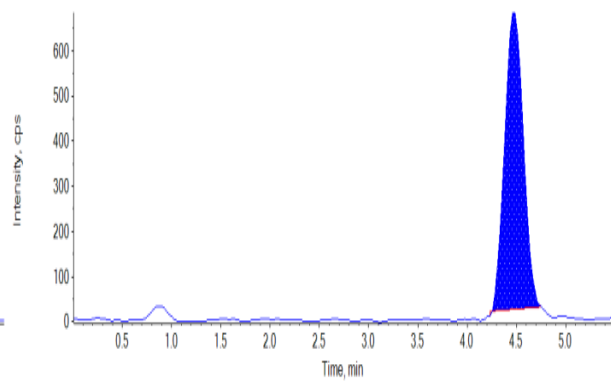

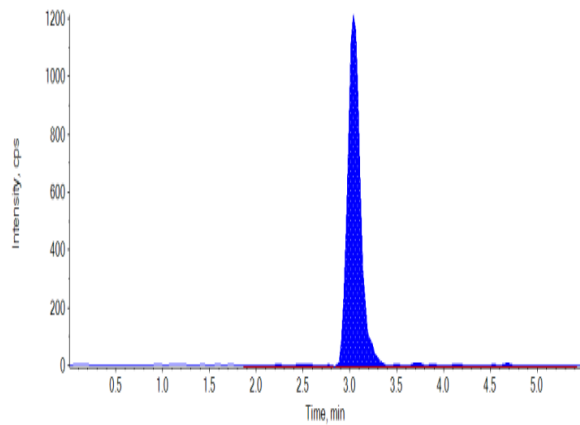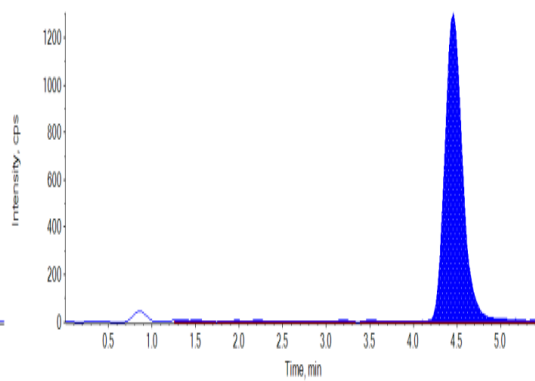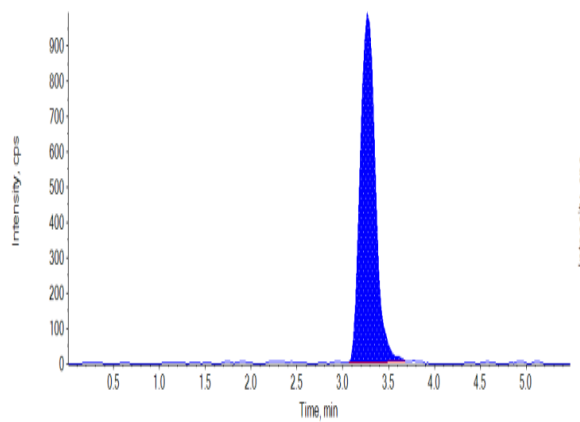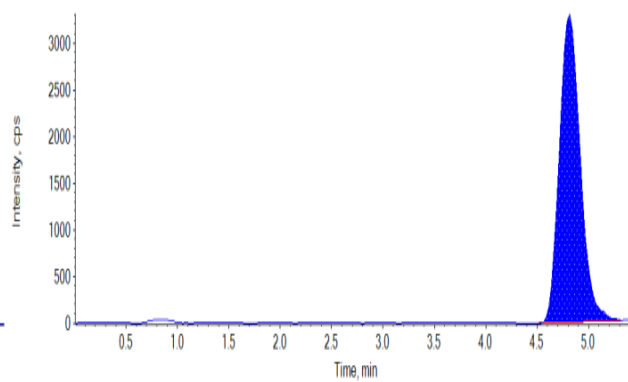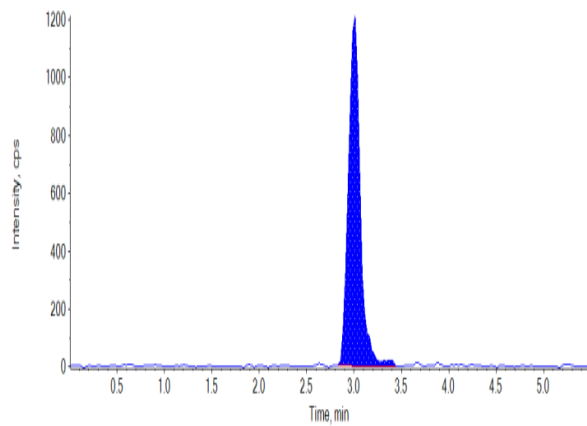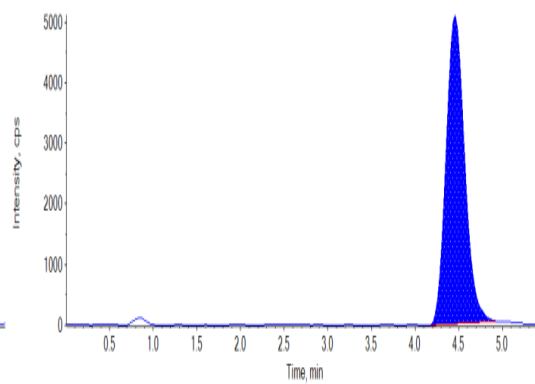

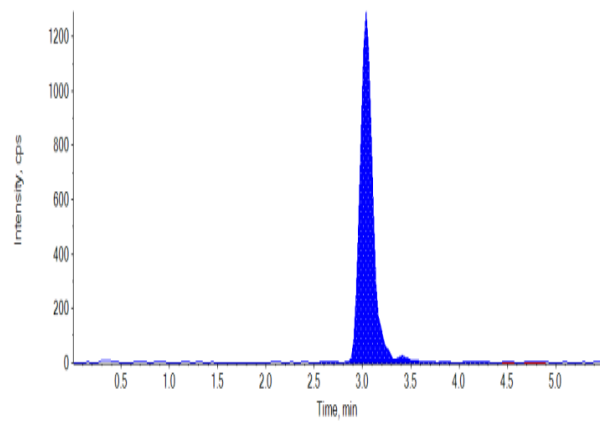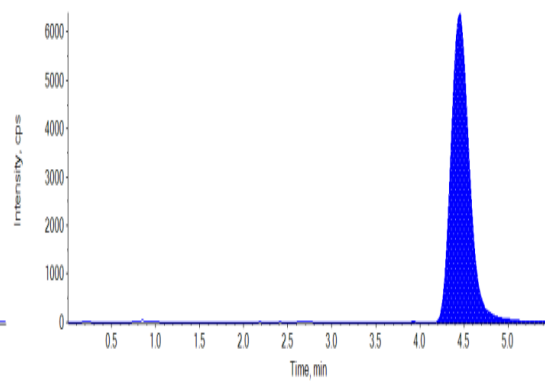

Supplement: Supplementary file 2 — Additional file 2. Chromatograms of increasing concentrations of calibration curve of canagliflozin. [file 13065_2019_597_MOESM2_ESM.pdf]
